# Syndecan-4 Inhibits the Development of Pulmonary Fibrosis by Attenuating TGF-β Signaling

**DOI:** 10.3390/ijms20204989

**Published:** 2019-10-09

**Authors:** Yoshinori Tanino, Xintao Wang, Takefumi Nikaido, Kenichi Misa, Yuki Sato, Ryuichi Togawa, Takaya Kawamata, Masami Kikuchi, Charles W. Frevert, Mishie Tanino, Tetsuhito Kojima, Yoko Shibata

**Affiliations:** 1 Department of Pulmonary Medicine, Fukushima Medical University School of Medicine, 1 Hikarigaoka, Fukushima 960-1295, Japan; xintaow@fmu.ac.jp (X.W.); taken@fmu.ac.jp (T.N.); kmisa@fmu.ac.jp (K.M.); yukisato@fmu.ac.jp (Y.S.); ryuichi@fmu.ac.jp (R.T.); kawamata@fmu.ac.jp (T.K.); k-masami@fmu.ac.jp (M.K.); shibatay@fmu.ac.jp (Y.S.); 2 The Center of Lung Biology, University of Washington School of Medicine, 850 Republican St, Seattle, WA 98109, USA; cfrevert@uw.edu; 3 Department of Pathology, Asahikawa Medical University Hospital, Midorigaoka-Higashi 2-1-1, Asahikawa, Hokkaido 078-8510, Japan; mtanino@asahikawa-med.ac.jp; 4 Department of Pathophysiological Laboratory Sciences, Nagoya University Graduate School of Medicine, 1-1-20 Daiko-Minami, Higashi-ku, Nagoya, Aichi 461-8673, Japan; kojima@met.nagoya-u.ac.jp

**Keywords:** syndecan-4, pulmonary fibrosis, TGF-β, fibroblasts, proteoglycan

## Abstract

Syndecan-4 is a transmembrane heparan sulfate proteoglycan expressed in a variety of cells, and its heparan sulfate glycosaminoglycan side chains bind to several proteins exhibiting various biological roles. The authors have previously demonstrated syndecan-4′s critical roles in pulmonary inflammation. In the current study, however, its role in pulmonary fibrosis was evaluated. Wild-type and syndecan-4-deficient mice were injected with bleomycin, and several parameters of inflammation and fibrosis were analyzed. The mRNA expression of collagen and α-smooth muscle action (α-SMA) in lung tissues, as well as the histopathological lung fibrosis score and collagen content in lung tissues, were significantly higher in the syndecan-4-deficient mice. However, the total cell count and cell differentiation in bronchoalveolar lavage fluid were equivalent between the wild-type and syndecan-4-deficient mice. Although there was no difference in the TGF-β expression in lung tissues between the wild-type and syndecan-4-deficient mice, significantly more activation of Smad3 in lung tissues was observed in the syndecan-4-deficient mice compared to the wild-type mice. Furthermore, in the in vitro experiments using lung fibroblasts, the co-incubation of syndecan-4 significantly inhibited TGF-β-induced Smad3 activation, collagen and α-SMA upregulation. Moreover, syndecan-4 knock-down by siRNA increased TGF-β-induced Smad3 activation and upregulated collagen and α-SMA expression. These findings showed that syndecan-4 inhibits the development of pulmonary fibrosis, at least in part, through attenuating TGF-β signaling.

## 1. Introduction

Idiopathic pulmonary fibrosis (IPF) is a chronic, progressive intractable fibrosing lung disease with a mean survival time of only 3–5 years from diagnosis. The annual incidence rate and prevalence of IPF in Japan are 2.5 and 11.0 per 100,000 population, respectively, and it is estimated that more than 13,000 patients are affected [1]. The mechanism of pulmonary fibrosis is complex, and various types of cells are involved. Pulmonary fibroblasts play an important role in pulmonary fibrosis by producing excessive extracellular matrix (ECM) and differentiating into myofibroblasts [2]. However, the full picture of the molecular pathophysiology of pulmonary fibrosis remains unclear.

Proteoglycans are glycoproteins consisting of a core protein with glycosaminoglycan (GAG) side chains. Several types of proteoglycans exist in the lung as components of extracellular matrices and were previously recognized only as the molecular glue that provided structural support to tissues. However, growing evidence suggests that proteoglycans have a variety of biological activities providing fine control of inflammation, wound healing, development, and homeostasis [3,4,5,6]. Heparan sulfate is the most abundant GAG in healthy lungs, and heparan sulfate proteoglycans (HSPGs) play a key role in tissue inflammation [7,8,9]. Syndecans are a family of HSPGs consisting of four isoforms. Syndecan-1, -2, and -3 are specifically expressed on the surfaces of epithelial cells or plasma cells, fibroblasts or endothelium, and nerve cells, respectively. On the other hand, syndecan-4 is expressed on a variety of cells, including alveolar macrophages, epithelial cells, endothelial cells, and fibroblasts [10,11,12]. Syndecans are also reported to be cleaved from cell surfaces by matrix metalloproteinase (MMP)-7, -9, or a disintegrin and metalloproteinase domain-containing protein-17 (ADAM-17), and exist as soluble forms [13,14,15,16]. The heparan sulfate GAG side chains of syndecans bind to various cytokines, chemokines, and growth factors, mediating their biological activities [8].

Our previous work shows that the treatment of syndecan-4-deficient (*Sdc4*KO) mice with intratracheal lipopolysaccharide (LPS) results in a significant increase in the recovery of neutrophils and CXC chemokines (KC, MIP-2) in bronchoalveolar lavage (BAL) fluid when compared to wild-type (WT) mice [6]. This study also found that serum syndecan-4 levels are higher in hospitalized bacterial pneumonia patients than in healthy individuals, and that syndecan-4 levels tend to increase over the course of pneumonia in patients with favorable prognosis [17]. These results showed that syndecan-4 has a protective role in acute lung inflammation. However, the role of syndecan-4 in pulmonary fibrosis remains to be clarified.

## 2. Results

### 2.1. Time Course of Syndecan-4 Expression in Lung Tissues

**Figure 1:**
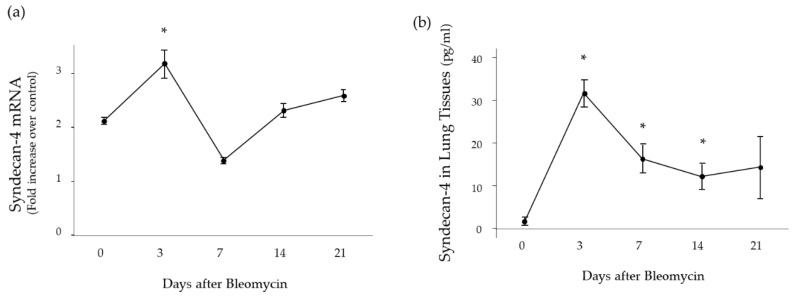
The expression of syndecan-4 in mouse lung tissue after intratracheal bleomycin instillation. (**a**) mRNA expression of syndecan-4 in the lung tissue of mice was significantly elevated at 3 days compared to baseline. (**b**) Syndecan-4 protein concentration in the lung tissue of mice was significantly elevated at 3, 7 and 14 days compared to baseline. The statistical differences between each group and Day 0 were compared using the Mann-Whitney *U* test. *, *p* < 0.05 vs. Day 0 (*n* = 3–6/each group).

To characterize the changes in the syndecan-4 expression, wild type (WT) mice were treated with bleomycin (BLM) and analyzed by quantitative real-time PCR and ELISA. Three days after the BLM treatment, the mRNA expression of syndecan-4 was significantly elevated compared to the baseline level (Figure 1a). Further, the protein concentration of syndecan-4 in lung tissue was significantly higher at 3, 7, and 14 days compared to the baseline level (Figure 1b).

### 2.2. Pulmonary Fibrosis in Syndecan-4 Deficient Mice

**Figure 2:**
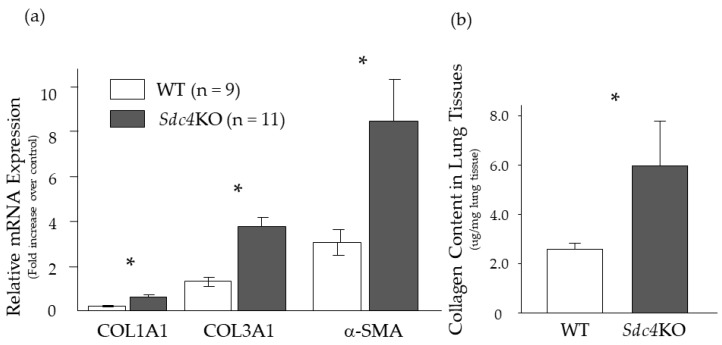
Pulmonary fibrosis after intratracheal bleomycin instillation. (**a**) mRNA expression of type I (COL1A1) and type III (COL3A1) collagen and α-SMA in the lung tissue was significantly higher in the syndecan-4-deficient (*Sdc4*KO; *n* = 11) mice than the wild-type (WT; *n* = 9) mice at 14 days after intratracheal bleomycin instillation. (**b**) Collagen content in lung tissues was significantly higher in *Sdc4*KO (*n* = 9) than in WT (*n* = 14) mice at 21 days. The statistical differences between WT and *Sdc4*KO mice were compared using the Mann–Whitney *U* test. * *p* < 0.05.

**Figure 3:**
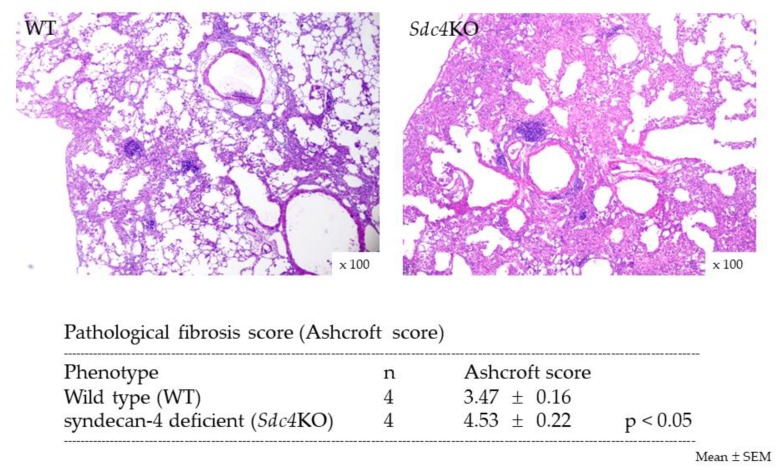
Histopathological findings at 21 days after intratracheal bleomycin instillation. Upper figures of hematoxylin and eosin staining were representatives of four mice. Pathological lung fibrosis score of the syndecan-4-deficient mice (*Sdc4*KO) was significantly higher compared to wild-type mice (WT) at 21 days after intratracheal bleomycin instillation. The difference was compared using the Mann-Whitney *U* test.

To identify the role of syndecan-4 in the development of pulmonary fibrosis, an intratracheal injection of BLM was performed in WT and syndecan-4 deficient (*Sdc4*KO) mice. At 14 days after BLM, the mRNA expression of type I (COL1A1) and III (COL3A1), and α-smooth muscle actin (α-SMA) was significantly higher in the *Sdc4*KO mice compared to the WT mice (Figure 2a). As the time point of 14 days might be too early for the evaluation of pulmonary fibrosis after BLM instillation in mice, the extent of pulmonary fibrosis was analyzed at 21 days. At this time, significantly higher collagen content was observed in the lung tissues (Figure 2b), as well as more severe fibrosis (Figure 3) in the *Sdc4*KO mice compared to the WT mice.

### 2.3. Bronchoalveolar Lavage Findings in Syndecan-4 Deficient Mice

**Figure 4:**
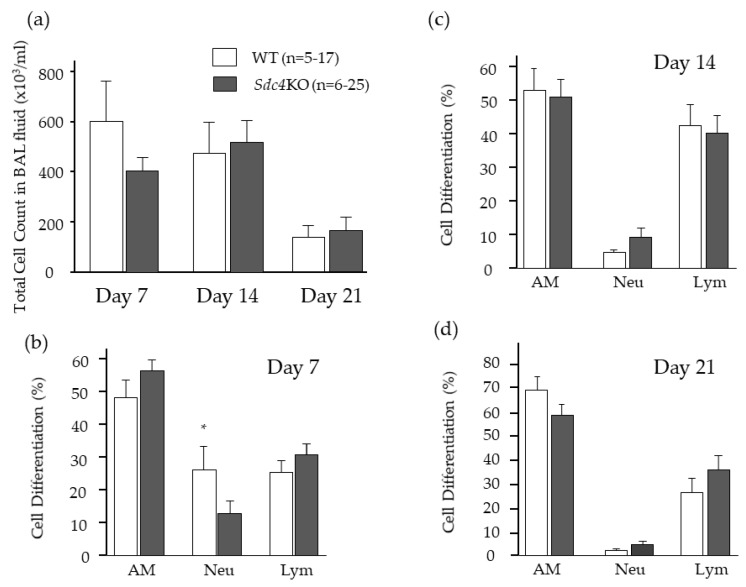
Bronchoalveolar lavage fluid findings after intratracheal bleomycin instillation. (**a**) Total cell count in BAL fluid did not differ between the wild-type (WT; *n* = 5–17) and syndecn-4-deficient (*Sdc4*KO; *n* = 6–25) mice at 7, 14 and 21 days after bleomycin instillation. (**b**-**d**) In addition, there was no difference regarding the cell differentiation in BAL fluid except neutrophils at seven days between the two groups. The difference was compared using the Mann–Whitney *U* test. AM: alveolar macrophages, Neu: neutrophils, Lym: lymphocytes. *, *p* < 0.05 vs. *Sdc4*KO.

The findings of bronchoalveolar lavage (BAL) fluid were analyzed to investigate the reason for the increased severity of pulmonary fibrosis in the *Sdc4*KO mice. At 7, 14 and 21 days after BLM, the total cell count in BAL fluid did not differ between the WT and *Sdc4*KO mice (Figure 4a). In addition, there was no difference regarding the cell differentiation in BAL fluid except neutrophils at seven days between the two groups (Figure 4b–d).

### 2.4. TGF-β Expression in Syndecan-4 Deficient Mice

**Figure 5:**
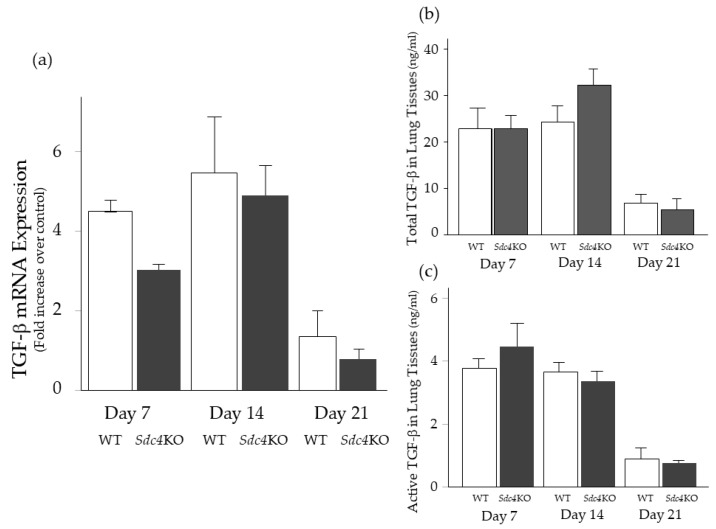
The expression of TGF-β in lung tissue after intratracheal bleomycin instillation. mRNA expression of TGF-β (**a**) and protein concentrations of total (**b**) and active (**c**) TGF-β in the lung tissue were not different between the wild-type (WT; *n* = 5–10) and syndecan-4-deficient (*Sdc4*KO; *n* = 6–11) mice at 7, 14 and 21 days after intratracheal bleomycin instillation. The difference was compared using the Mann-Whitney *U* test.

For further analysis, this study analyzed the expression of TGF-β, which plays critical roles in pulmonary fibrosis in lung tissues. The mRNA expression of TGF-β as well as the total and active concentrations of TGF-β in the lung tissues did not differ between the two groups, at 7, 14 and 21 days after BLM (Figure 5).

**Figure 6:**
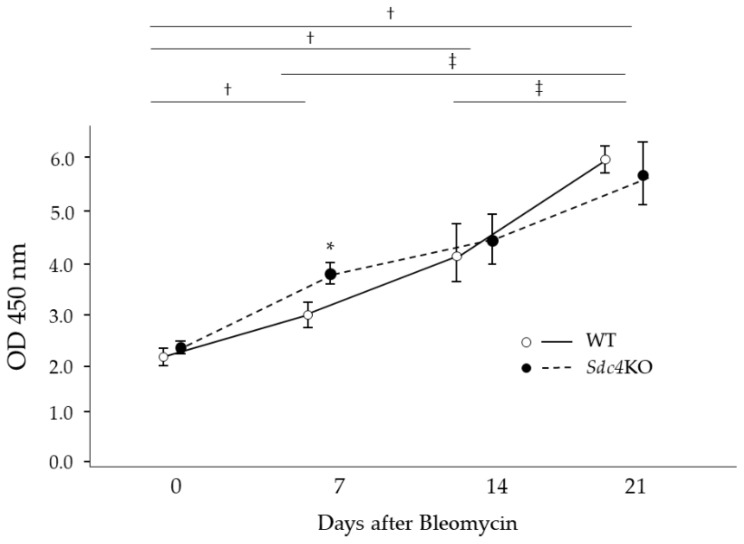
Levels of phosphorylated Smad3 in lung tissues. Levels of phosphorylated Smad3 in lung tissues were higher in the syndecan-4-deficient (*Sdc4*KO) mice compared to the wild-type (WT) mice at 7 days after intratracheal bleomycin instillation. The difference were compared using the Mann-Whitney *U* test as well as the ANOVA test with Fisher’s least significant difference test as a post hoc test. *, *p* < 0.05 vs. WT at 7 days after bleomycin instillation, †, *p* < 0.05 vs. WT and *Sdc4*KO without bleomycin instillation, ‡, *p* < 0.05 versus WT and *Sdc4*KO at 21 days after bleomycin instillation (*n* = 3–7/each group).

However, the phosphorylation of Smad3 in lung tissues was significantly increased in the *Sdc4*KO mice compared to the WT mice at seven days (Figure 6).

### 2.5. Effect of Syndecan-4 on TGF-β-induced Upregulation of Collagen and α-SMA in Lung Fibroblasts

**Figure 7:**
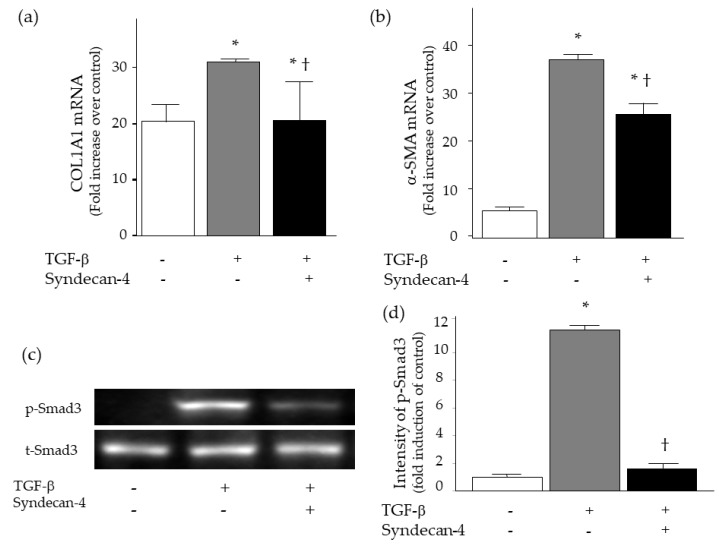
Expression of collagen, α-SMA and Smad3 in WI-38 lung fibroblasts after TGF-β stimulation with or without co-incubation of syndecan-4. (**a**,**b**) The co-incubation of recombinant syndecan-4 significantly decreased the mRNA expression of TGF-β-induced collagen and α-SMA upregulation at 24 h (*n* = 5–9 per each condition). (**c**,**d**) TGF-β-induced phosphorylation of Smad3 was attenuated by the co-incubation of recombinant syndecan-4 at 15 min. (**c**) were representatives of three separate experiments, and (**d**) showed intensity of p-Smad3 evaluated by densitometric analysis. The difference was compared using the ANOVA test and Fisher’s least significant difference test as a post hoc test. *, *p* < 0.05 vs. TGF-β (−) and syndecan-4 (−), †, *p* < 0.05 versus TGF-β (+) and syndecan-4 (−).

To evaluate the role of syndecan-4 in pulmonary fibrosis, in vitro experiments were conducted using WI-38 lung fibroblasts. Lung fibroblasts were stimulated with TGF-β with or without co-incubation of recombinant syndecan-4. At 24 h, the co-incubation of recombinant syndecan-4 significantly decreased the mRNA expression of TGF-β-induced collagen and α-SMA upregulation (Figure 7a,b). In addition, TGF-β-induced phosphorylation of Smad3 was attenuated by the co-incubation of recombinant syndecan-4 at 15 min (Figure 7c,d).

### 2.6. Effect of Syndecan-4 Knock-Down on TGF-β-Induced Upregulation of Collagen and α-SMA in Lung Fibroblasts

**Figure 8:**
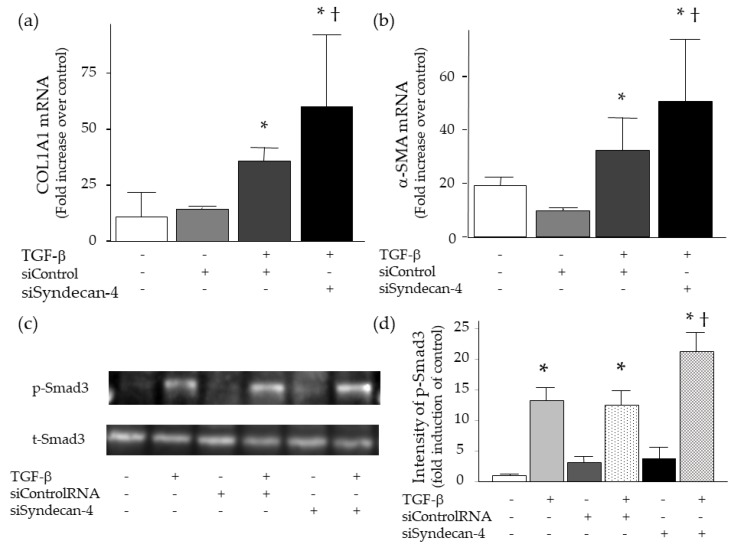
Expression of collagen, α-SMA and Smad3 in WI-38 lung fibroblasts after TGF-β stimulation with or without knockdown of syndecna-4. (**a**,**b**) TGF-β-induced type I collagen (COL1A1) and α-SMA upregulation significantly increased 24 h after knockdown of syndecan-4 at 24 h (*n* = 5–9 per each condition). (**c**,**d**) TGF-β-induced phosphorylation of Smad3 was increased by knockdown of syndecan-4 at 15 min. Figures (**c**) were representatives of three separate experiments, and figure (**d**) showed intensity of p-Smad3 evaluated by densitometric analysis. The difference was compared using the ANOVA and Fisher’s least significant difference test as a post hoc test. *, *p* < 0.05 vs. TGF-β (−), siControl (−) and siSyndecan-4 (−), †, *p* < 0.05 vs. TGF-β (+), siControl (−) and siSyndecan-4 (+). siControl: control siRNA, siSyndecan-4: siRNA for syndecan-4, t-Smad3: total Smad3, p-Smad3: phosphorylated Smad3.

For further evaluation of the role of syndecan-4 on pulmonary fibrosis, the effect of syndecan-4 knock-down on TGF-β-induced collagen and α-SMA upregulation in lung fibroblasts was analyzed. This study first confirmed that transfection of syndecan-4 siRNA induced a significant decrease in syndecan-4 mRNA at 24 h (97.4% reduction compared to the control). Consistent with the results of syndecan-4 co-incubation, TGF-β-induced collagen and α-SMA upregulation significantly increased 24h after knockdown by syndecan-4 siRNA (Figure 8a,b). These effects were accompanied by increased phosphorylation of Smad3 at 15 min (Figure 8c,d).

### 2.7. Binding of TGF-β to Syndecan-4

**Figure 9:**
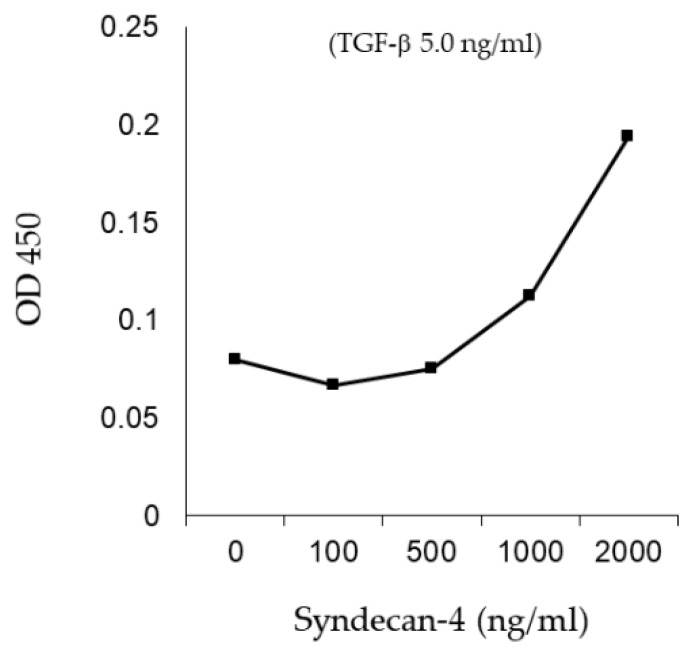
Binding of recombinant TGF-β and syndecan-4. In vitro binding assay showed a dose-dependent binding of recombinant TGF-β to recombinant syndecan-4.

As proteoglycans, such as syndecan-4, exhibit several biological roles by binding to several proteins via its HSPG side chains, this study evaluated whether TGF-β binds to syndecan-4 *in vitro*. Figure 9 shows that recombinant TGF-β binds to recombinant syndecan-4 in a dose-dependent manner.

## 3. Discussion

The purpose of this study was to identify the role of syndecan-4 in pulmonary fibrosis. The treatment of mice with intratracheal BLM instillation resulted in an increase in syndecan-4 in the lungs, and the results from the in vivo experiments using *Sdc4*KO mice showed that the lack of this HSPG resulted in a significant increase in pulmonary fibrosis with more Smad3 activation following treatment with BLM. Moreover, the in vitro experiments showed that TGF-β-induced collagen and α-SMA upregulation as well as Smad3 activation was attenuated with recombinant syndecan-4 co-incubation and enhanced by syndecan-4 knockdown in lung fibroblasts. Furthermore, the in vitro binding assay showed the binding of TGF-β with syndecan-4. These results show that syndecan-4 regulates TGF-β signaling by sequestering active TGF-β from TGF-β receptor and inhibits the development of pulmonary fibrosis.

Syndecan-4 is a HSPG consisting of a core protein with heparan sulfate side chains, and is expressed in a variety of cells, such as alveolar macrophages, epithelial cells, endothelial cells and fibroblasts [10,11,12]. Although proteoglycans like syndecan-4 had been considered to be a molecular glue, their significant biological roles have now been recognized. Syndecans are reported to regulate cell migration, proliferation, differentiation, adhesion and apoptosis, as well as play critical roles in inflammation and wound healing [3,4,5,6]. GAG side chains bind to several proteins such as chemokines, growth factors and integrin, regulating their bioactivities. Syndecan-4 exists on cell membranes (membrane-bound form), is reported to act as a co-receptor for several mediators via its heparan sulfate GAGs, and may concentrate heparin-binding proteins on the cell surface. In addition, syndecan-4 exists as a soluble ectodomain form after proteolytic cleavage by proteases, such as MMPs and ADAM-17 [13,14,15,16]. A soluble form of syndecan-4 can bind to several proteins and retain a similar bioactivity as a membrane-bound form [6,18,19]. These properties contribute to the significant roles of syndecan-4 in a variety of diseases [17,20,21,22,23].

There is limited evidence on the role of syndecan-4 in pulmonary fibrosis. Jiang et al. analyzed BLM-induced pulmonary fibrosis using *Sdc4*KO, and showed that CXCL10 needs binding to syndecan-4 GAG side chains for its anti-fibrotic activity [24]. In addition, Santosa et al. reported that intraperitoneal and intratracheal instillation of recombinant syndecan-4 decreased the amounts of soluble collagen in bleomycin-instilled lungs by an increase in bronchial progenitor cells at eight days [25]. Moreover, it has been reported that syndecan-4 is overexpressed in the fibroblasts of patients with diffuse systemic sclerosis, and reduces the ability of contract TGF-β-induced collagen matrix and ERK activation in dermal fibroblasts [26]. These results suggest that syndecan-4 has anti-fibrotic activity in pulmonary fibrosis. This anti-fibrotic activity is also reported to be in the fibrosis of other tissues, such as the kidney [27,28] and the heart [29].

However, conflicting results have been reported as well. Lipphardt et al. recently reported that soluble syndecan-4 ectodomain is responsible for fibrosis in the renal unilateral ureteral obstruction model [14]. Syndecan-4 has been shown to be important for the differentiation of cardiac fibroblasts into myofibroblasts, and is involved in the development of pressure-overloaded cardiac fibrosis [30]. Regarding syndecans, soluble syndecan-1 ectodomain has been reported to cause neutrophil chemotaxis and aberrant wound healing in asbest and BLM-induced pulmonary fibrosis models, showing the pro-fibrotic activity of syndecan-1 [31]. Syndecan-2 has been revealed to bind to TGF-β and increase its signaling [32], and is overexpressed in the fibrotic lung tissues of patients with systemic sclerosis and IPF [33]. The inhibition of syndecan-2 has also been reported to abolish TGF-β-dependent adhesion with decreased Smad2 activation in fibrosarcoma cells [34]. Moreover, it has been reported that syndecan-4 enhances platelet-derived growth factor-BB activity in diabetic wound healing [35], and promotes TGF-β-induced epithelial mesenchymal transition in A549 lung alveolar epithelial cells, showing the pro-fibrotic activity of syndecan-4 [36]. Taken together, these data from published reports do not show consistent results regarding the activity of syndecans, especially syndecan-4 for tissue fibrosis.

To determine the role of syndecan-4 in pulmonary fibrosis, mice that were deficient in syndecan-4 were studied. Our results show that the *Sdc4*KO mice had increased pulmonary fibrosis following treatment with BLM. These results are consistent with previous reports, such as the work of Jiang et al., who reported that syndecan-4 inhibited fibroblast recruitment and subsequent fibrosis by binding to CXCL10 [24]. In the report by Santosa et al., the mechanism of inhibition of pulmonary fibrosis by recombinant syndecan-4 instillation was shown to be increased in bronchial progenitor cells [25]. In the present report, it was shown that there was more severe pulmonary fibrosis and activation of Smad3 in the lung tissues of *Sdc4*KO mice when compared to WT mice. In addition, the TGF-β-induced upregulation of collagen and α-SMA was attenuated by recombinant syndecan-4 and enhanced by syndecan-4 knockdown in lung fibroblasts. These changes in collagen and α-SMA expression were accompanied by changes in Smad3 activation. As this study also showed the binding of syndecan-4 with TGF-β in vitro, the anti-fibrotic activity of syndecan-4 is, at least in part, due to inhibition by the binding of syndecan-4 and TGF-β.

In the current study, several mechanistically relevant questions of interest were not addressed. Lung fibroblasts were analyzed because the cells played a critical role in pulmonary fibrosis and the increase in syndecan-4 had already been demonstrated in the cells from BLM-instilled lung tissues [24]. However, the possibility that other types of cells are involved in the pathogenesis of pulmonary fibrosis cannot be excluded, because syndecan-4 exists in a variety of cells. In LPS-induced lung inflammation, the focus was on the role of syndecan-4 in macrophages and epithelial cells and showed the important roles of these cells in lung inflammation [6]. The discrepancy regarding the role of proteoglycan on growth factor signaling was not studied. In the present study, syndecan-4 inhibited TGF-β signaling. However, previous studies have demonstrated that syndecans act as co-receptors for several mediators via their heparan sulfate GAGs and concentrate heparin-binding proteins on the cell surface [3,37,38]. The role of syndecan-4 has been considered to facilitate the binding of heparin-binding proteins to their original receptors, leading to the acceleration of signals [39]. Lu et al. reported that the binding of TGF-β to heparan sulfate at the cell membrane helps bring TGF-β to close proximity to its signaling receptors, and increases the local concentration of TGF-β [40]. Our results were not consistent with the previous data. Syndecan-4 exists as two forms: A membrane-bound form and a soluble ectodomain form. There are some studies reporting that a soluble ectodomain form of proteoglycans competes with a membrane-bound form in the binding of heparin-binding proteins, resulting in the inhibition of activities of heparin-binding proteins. This hypothesis is consistent with our results that co-incubation of recombinant syndecan-4 inhibited the upregulation of collagen and α-SMA in lung fibroblasts and CXCL8 in lung epithelial cells [6]. However, further study is required to determine the exact reason why syndecan-4 knockdown in lung fibroblasts enhanced TGF-β signaling. The lack of syndecan-4, which traps heparin-binding proteins, such as TGF-β, may facilitate the binding of the proteins to their original receptors. However, this hypothesis is inconsistent with the findings of previous studies. In addition, this study did not evaluate other proteoglycans, such as syndecan-1, and -2. However, it is not possible to evaluate all proteoglycans in a single study because there are so many different types. Even in HSPG, more than ten PGs were identified [7,41]. To clarify the exact roles of PGs, such as syndecan-4, further intensive studies are necessary.

In summary, the results of the current study show that syndecan-4 increased in response to the intratracheal instillation of BLM and the mice lacking Sdc4 had an increased pulmonary fibrotic response when treated with intratracheal BLM. Finally, the co-incubation with recombinant syndecan-4 inhibits and the knockdown of syndecan-4 enhances the fibrotic properties of TGF-β in lung fibroblasts in vitro. Taken together, this study concludes that syndecan-4 inhibits the development of pulmonary fibrosis, at least in part, through attenuating TGF-β signaling in the lungs.

## 4. Materials and Methods

### 4.1. Reagents

Bleomycin hydrochloride (kindly donated by Nippon Kayaku, Tokyo, Japan), Power SYBR Green PCR master mix (Applied Biosystems, Foster City, CA, USA), WI-38 cells (ATCC, Manassas, VA, USA), human recombinant TGF-β1 (Sigma-Aldrich, St. Louis, MO, USA), human syndecan-4 (R&D, Minneapolis, MN, USA).

### 4.2. Animal Protocols

The Animal Research Committee of Fukushima Medical University approved all animal experiments (approved number: 167, date: 5 January 2012). The C57BL/6 and *Sdc4*KO mice (Dr. T. Kojima) were housed in specific pathogen-free facilities. The *Sdc4*KO mice were totally lack of syndecan-4 in the bodies. The single intratracheal injection of BLM (2.5 μg/g), euthanasia at specified times, BAL, and the total and differential cell counts were performed as previously described [42].

### 4.3. Time Course of Syndecan-4 Expression

The mRNA levels of syndecan-4 levels in lung tissues were analyzed by quantitative real-time PCR using the following primers: Fwd: 5′-CGAGAGACTGAGGTCATCGAC-3′, Rev: 5′-GCGGTAGAACTCATTGGTGG-3′ as previously described [6]. The protein concentrations of syndecan-4 in lung tissues were analyzed by ELISA using the commercial kit (Wuhan Huamei Biotech, Houston, TX, USA) as previously described [17].

### 4.4. Isolation of RNA

RNA was isolated with the Absolute RNA Miniprep Kit (Stratagene, La Jolla, CA, USA). Genomic DNA was digested with DNAse I (Ambion, Austin, TX, USA) and RNA was reverse transcribed with the SuperScript III First-Strand Synthesis System (Invitrogen, Carlsbad, CA, USA).

### 4.5. Measurement of mRNA

Quantitative PCR was performed using Power SYBR Green PCR master mix and an ABI PRISM 7000 (Applied Biosystems, Foster City, CA, USA). The threshold cycle (*C*_t_) was calculated using threshold cycles for the target genes and GAPDH. The relative mRNA expression was expressed as fold increase over the values obtained from RNA from normal lungs or human reference total RNA (Stratagene, La Jolla, CA, USA).

### 4.6. Measurement of Collagen Content

The total soluble collagen content of lung tissue was determined using a Sircol Collagen Assay Kit (Biocolor, Carrickfergus, UK), as previously described [43]. Briefly, the stored frozen lung tissues were homogenized in 0.5 M acetic acid containing 0.1 mg/mL pepsin, then incubated and stirred over night at 4 °C. The collagen content was measured according to the manufacturer’s instructions.

### 4.7. Pathological Evaluation of Lung Sections

A pathological evaluation was performed as previously described. Briefly, the lungs were excised and fixed by inflation at 25 cm of H_2_O with a phosphate buffer (10mM, pH 7.4) containing 10% formalin for 24 h and then embedded in paraffin. A 5-μm-thick tissue section was prepared and stained with hematoxylin and eosin. Lung fibrosis severity was semi-quantitatively assessed from grade 0 to 8, according to the methods previously described [42,44]. The score was assessed blindly by two pulmonologists.

### 4.8. Measurement of TGF-β

The measurement of TGF-β in lung tissue was measured by ELISA for murine TGF-β (R&D, Minneapolis, MN, USA) according to the manufacture’s protocol. To measure the concentrations of the total and active TGF-β, all samples were assayed both with and without the acidification and neutralization to convert the latent form to the active form. Briefly, a 50 μL sample was mixed with 10 μL of 1 N HCl and, after 10 min at room temperature, neutralized with 10 μL of 1.2 N NaOH/0.5 M HEPES.

### 4.9. Measurement of Phosphorylated SMAD3

Phosphorylated SMAD3 of lung tissue was determined with a SMAD3 (pS423/S425) in vitro SimpleStep ELISA Kit (ab186038, Abcam, Cambridge, UK), according to the manufacturer’s protocol. The SimpleStep ELISA employs a labeled capture and detector antibody, which immunocaptures the sample analyte in solution. This entire complex (capture antibody/protein/detector antibody) is in turn immobilized in the well by immunoaffinity via the anti-tag antibody. The samples or standards are added to the wells, followed by the antibody mix. After incubation, the wells are washed to remove unbound material and the TMB substrate is then added. The reaction is stopped by the addition of Stop Solution. A signal is generated proportionally to the amount of bound analytes, and the intensity is measured at 450 nm.

### 4.10. Cell Culture

Human lung fibroblasts, WI-38 cells were cultured in MEME medium supplemented with 10% FBS (Gibco by life technologies, Grand Island, NY, USA), 100 IU/mL penicillin and 100 μg/mL streptomycin (Sigma-Aldrich). After reaching 80% confluence, the cells were isolated, counted and cultured in MEME medium for 24 h. Recombinant TGF-β1 (1.0 ng/mL) was added to the wells with or without 500 ng/mL of recombinant syndecan-4, and the cells were incubated and harvested at specified times.

### 4.11. Knock-Down of Syndecan-4

Small inhibitory RNAs (siRNAs) were obtained from Thermo Scientific (ON-TARGET plus SMART human SDC4 and ON-TARGET plus Non-Targeting siRNA, Waltham, MA, USA). The transfection of siRNAs was performed according to the manufacturer’s protocol. The WI-38 cells were incubated in growth medium for 24 h, and a final concentration of 100 nM siRNA was added to the cells. Lipofectamine RNAiMAX (Invitrogen) was used as a transfection medium. After 24 h, the cells were washed and incubated with or without TGF-β1 for specified times.

### 4.12. Western Blot Analysis

Briefly, WI-38 cells were lysed and sonicated by adding ice-cold lysis buffer with protease inhibitor (Roche Diagnostics, Mannheim, Germany), followed by centrifugation. Equal amounts of protein were electrophoresed in 12% SDS-PAGE gel, then transferred onto a Hybond-ECL nitrocellulose membrane (Amersham Biosciences, Freiburg, Germany). The proteins were probed with Smad3 and phospho-Smad3 antibodies (Cell Signaling Technology, Danvers, MA, USA) followed by peroxidase conjugated anti-rabbit IgG (Pierce Biotechnology, Rockford, IL, USA). The blot was developed with Super Signal West Femto Maximum Sensitivity Substrate (Pierce Biotechnology) and exposed to X-ray film (FUJIFILM, Tokyo, Japan). Densitometry data from each Western blot were taken individually by ImmageQuant LAS 4000 (GE Healthcare Life Science, Pittsburgh, PA, USA) for analysis with ImageQuant TL (GE Healthcare Life Science).

### 4.13. In Vitro Binding Assay

Binding of syndecan-4 and TGF-β was analyzed as previously described [18]. Briefly, 96-well ELISA plates were coated with dilute recombinant syndecan-4, 0, 100, 500 1000 or 2000 ng/mL (overnight at 4 °C). The following incubation and washing steps were performed at room temperature: (a) saturation in block solution (0.2% I-Black and 0.1% Tween 20, 1 h); (b) three washes (PBS with 0.05% Tween-20); (c) incubation with TGF-β (5 ng/mL in I-Block, 100 μL, 2 h); (d) three washes, (e) incubation with biotinylated anti-TGF-β antibody (BAF240, R&D) (0.5 ug/mL in block buffer, 100 μL, 1 h); (f) three washes; (g) add Streptavidin Poly-HRP detection (100 μL, 1 h), (h) three washes; (i) incubation with TMB substrate solution (100 μL, ~20 min) and termination (1M phosphoric acid, 100 μL). Absorbance was taken at 450 nm with a microplate reader.

### 4.14. Statistical Analysis

The data are expressed as the means ± standard error, unless otherwise stated. The Mann–Whitney *U* test was used to compare two groups, while ANOVA was used to compare multiple groups. Fisher’s least significant difference test was used for post hoc analysis. For all analysis, *p* < 0.05 was considered statistically significant.
